# A continuous wavelet transform-enhanced transformer model for spectroscopic detection of bacterial wilt in tomato at early infection stages

**DOI:** 10.3389/fpls.2026.1911300

**Published:** 2026-09-03

**Authors:** Xiaoya Ru, Tianzhi He, Huaiheng Cai, Ran Liu, Jiaming Si, Yunming Xu, Yimin Zhou, Zhujun Zhu, Tao Ji

**Affiliations:** 1 College of Horticulture, Zhejiang Agriculture and Forestry University, Hangzhou, Zhejiang, China; 2 Key Laboratory of Quality and Safety Control for Subtropical Fruit and Vegetable, Ministry of Agriculture and Rural Affairs, Hangzhou, Zhejiang, China; 3 College of Optical, Mechanical and Electrical Engineering, Zhejiang Agriculture and Forestry University, Hangzhou, Zhejiang, China; 4 Agricultural College, Shihezi University, Shihezi, Xinjiang, China

**Keywords:** continuous wavelet transform, deep learning, early detection, hyperspectral imaging, tomato bacterial wilt

## Abstract

**Introduction:**

Tomato (*Solanum lycopersicum L*.) bacterial wilt is a highly destructive soil-borne disease whose early-stage infections are asymptomatic or visually indistinguishable, limiting the effectiveness of conventional diagnostic methods.

**Methods:**

This study developed a Continuous Wavelet Transform-enhanced Transformer framework (CWT-STNet) for early-stage detection of bacterial wilt using hyperspectral data collected from tomato canopies under controlled inoculation conditions. Infection severity was categorized into grades 0 to 4, with levels 1–2 defined as the early infection stage. Raw spectral data were fused with multi-scale wavelet features extracted using Continuous Wavelet Transform (CWT), and the effects of different scale combinations on model performance were systematically evaluated. CWT-STNet was compared with one-dimensional convolutional neural networks (1D-CNNs), random forests (RF), and support vector machines (SVM). SHapley Additive exPlanations (SHAP) were further used to interpret model predictions and identify disease-sensitive spectral features.

**Results:**

The proposed CWT-STNet framework achieved an overall accuracy of 96.77% across all infection levels and an early-stage detection accuracy of 93.60%. In comparison, models relying only on raw spectra achieved 84.15% accuracy in distinguishing early disease severity. Incorporating CWT-derived multi-scale features improved the performance of all evaluated models, while ablation analysis confirmed the importance of combining raw spectra with multi-scale wavelet features. SHAP analysis identified spectral features around 869.0 nm and the 1160–1190 nm region as particularly important for early bacterial wilt detection.

**Discussion:**

The identified spectral regions were associated with physiological and structural changes related to bacterial wilt infection, including alterations in plant water status, leaf cellular structure, and pigment-related responses. These findings demonstrate that the proposed CWT-STNet framework provides a robust, interpretable, and non-destructive approach for early detection of tomato bacterial wilt and has potential for precision disease monitoring.

## 1 Introduction

Tomato bacterial wilt (BW), which is induced by the soil-borne pathogen *Ralstonia solanacearum*, represents a serious vascular disorder that threatens tomato cultivation worldwide, with yield losses exceeding 90% under suitable environments (Ray et al., 2026). The pathogen of BW invades the xylem vessels of host plants and rapidly produces compounds, which obstruct water transport and ultimately results in wilting and death of the plant (Zhao et al., 2025). However, in the early stages of plant’s infection before obvious symptoms appear, the pathogen of BW begins to disrupt the host’s water conduction system. It can cause slight water shortages, fluctuations in stomatal behavior, and mild changes in pigment metabolism (Chen et al., 2020). These subtle physiological responses, though insufficient to form visible symptoms, reflect a potential disruption of the plant’s normal physiological functions. Although laboratory techniques such as polymerase chain reaction, *in situ* hybridization, and serology provide highly reliable detection (Cope-Arguello et al., 2026; Peng et al., 2025), their destructive nature and time-consuming procedures limit large-scale field application. Given the rapid spread of BW, there is an urgent need to develop timely and efficient early monitoring technologies to rapidly detect infected tomato plants in the early stages. This would enable the prompt implementation of appropriate control measures, reduce the risk of secondary infection, limit further disease transmission, and ultimately support the stability and sustainability of tomato production (Wang et al. 2023a).

Hyperspectral imaging is a valuable tool for monitoring plant health, because it can capture continuous spectral signatures that are closely related to the biochemical composition and internal structure of leaves (Mahlein et al., 2018; Nagasubramanian et al., 2018). Changes caused by plant pathogen infection, including alterations in chlorophyll concentration, water content and tissue structure, can be reflected in the reflection spectrum, which makes it possible to detect stress conditions in plants before visible symptoms appear. Previous studies have confirmed that hyperspectral technology can favorably detect fluctuations in plant physiological state caused by various pathogens such as bacteria and fungi, demonstrating great potential in early-stage disease identification (Ong et al., 2025; Wang et al., 2023b). In particular, spectral regions including the red edge and near-infrared bands are widely recognized as responsive to chlorophyll dynamics and leaf structural changes to in response plant stress (Xie et al., 2024). However, the intrinsic properties of hyperspectral data—high dimensionality, severe redundancy, and strong inter-band correlation—substantially complicate feature extraction and band selection. Consequently, disease-sensitive signals are frequently obscured by massive amounts of redundant data and background noise. This challenge is particularly acute in the early diagnosis of tomato bacterial wilt: canopy spectral responses induced by *Ralstonia solanacearum* infection are extremely subtle, and early physiological alterations (e.g., shifts in water status and chlorophyll content) closely mimic those triggered by other biotic and abiotic stresses (Sapes et al., 2024; Zarco-Tejada et al., 2021). Compounded by environmental fluctuations and inter-plant variability, these combined factors severely constrain the detection efficacy of hyperspectral technology in this specific scenario (Zhou et al., 2025).

To address these challenges, deep learning models, such as 1D-CNN, attention-enhanced architectures, and multilayer perceptions, have been widely adopted in hyperspectral data analysis to automatically extract hierarchical spectral-spatial features and reduce the burden of manual feature engineering. However, despite the powerful nonlinear mapping capabilities of deep learning, its integration with hyperspectral imaging in agricultural disease diagnosis is still constrained by several bottlenecks: First, the model relies on large-scale, finely labeled data to suppress overfitting, but obtaining accurate labels for asymptomatic infections in early pathology is expensive and destructive (e.g., PCR verification), resulting in a severe shortage of training samples. Second, deep models lack physical interpretability, making it difficult to establish a correlation between learned features and actual physiological changes in plants (e.g., vascular bundle blockage or water deficit). In the early detection of bacterial wilt in tomatoes, the model’s sensitivity to background noise, weak cross-environment generalization, and susceptibility to interference from light and canopy geometric variations further exacerbate the detection risk, severely limiting the robustness and practical application effectiveness of this method in this scenario.

Continuous wavelet transform (CWT), a signal processing method capable of improving spectral resolution, signal-to-noise ratio, and characteristic peak identification, is highly sensitive to local spectral changes and can automatically identify peak positions, widths, and symmetries (Lv et al., 2023). By performing multi-scale decomposition on the spectral signal, CWT can simultaneously provide local spectral information in both the wavelength domain (time domain) and the scale domain (frequency domain), making it particularly suitable for identifying weak absorption features related to plant physiological changes and effectively suppressing background noise (Cheng et al., 2010). Therefore, it is a promising approach to combining hyperspectral data with CWT to build a multi-scale spectral feature representation method, which can overcome the shortcomings of traditional single-scale methods (such as derivative spectroscopy) in extracting weak and overlapping disease information.

Although CWT can effectively extract weak local spectral features, physiological changes caused by vascular diseases often exhibit complex long-range correlations across multiple spectral bands. Traditional machine learning models such as Random Forests (RF), Support Vector Machines (SVM) or Convolutional Neural Networks (CNN) struggle to simultaneously capture these cross-band global dependency relationships (Liu et al., 2022). Therefore, this study further introduces a Transformer network model, utilizing its self-attention mechanism to globally model the multi-scale spectral features after CWT processing, aiming to more accurately identify the early stages of infection in tomato plants. The intrinsic relationship between the disease and spectral characteristics can guide the optimization of model structure and he development of a reliable early diagnosis system. To elucidate the intrinsic mechanism between diseases and spectral features and identify the spectral bands most sensitive to early infection, the game theory-based SHapley Additive exPlanations (SHAP) method was employed to interpret the Transformer model (Zarco-Tejada et al., 2021). By calculating the marginal contribution of each spectral feature (or wavelength-scale) after CWT transformation to the model output, the key spectral bands contributing most to early detection of BW can be quantitatively identified. This analysis not only compensates for the lack of interpretability of the Transformer model but also provides interpretable spectral evidence for the early detection of bacterial wilt at the field scale.

In this study, a Continuous Wavelet Transform-enhanced Transformer Network framework (namely, CWT-STNet model) was proposed to analyze hyperspectral data collected from tomato canopies for early-stage detection of bacterial wilt caused by *Ralstonia solanacearum*. The objectives are: (1) to perform multi-scale decomposition of hyperspectral data of tomato plants collected from day 1 to day 6 after inoculation with *Ralstonia solanacearum* using CWT; (2) to develop a CWT-STNet model capable of global feature learning and evaluate its performance. The developed model is then systematically compared with traditional models (CNN, SVM, and RF) to evaluate its effectiveness in early detection of bacterial wilt in tomato. (3) to conduct SHAP-based model interpretability analysis and spatial mapping of disease stress based on the CWT-STNet model. This study provides an accurate and interpretable early detection framework for tomato BW, offering a promising technical foundation for non-destructive field diagnosis and precision disease supervision.

## 2 Materials and methods

**Figure 1:**
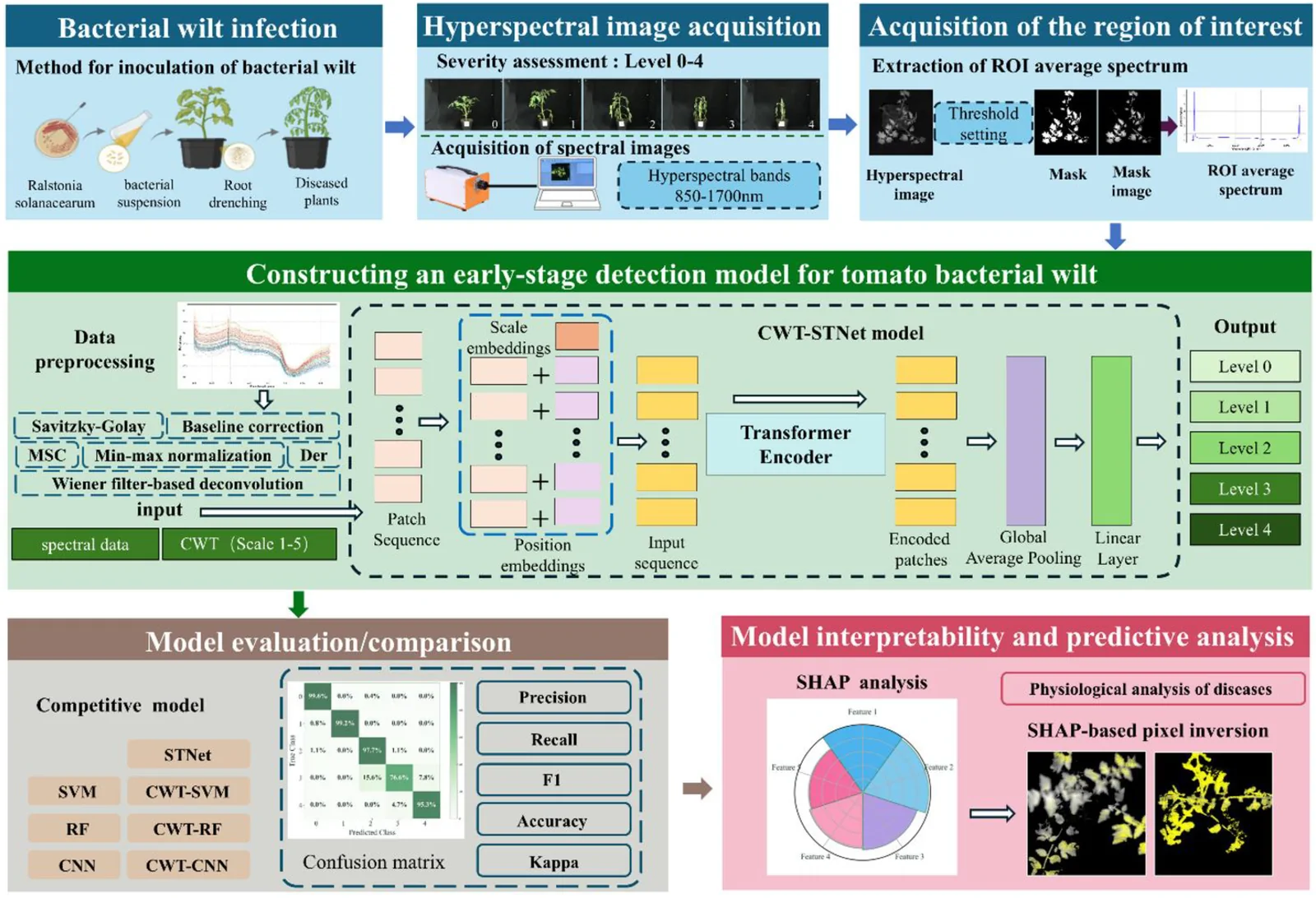
Framework of the proposed CWT-STNet model for early-stage detection of tomato BW.

This study used tomato plants artificially inoculated with *Ralstonia solanacearum* to collect canopy hyperspectral images (850–1700 nm) from day 1 to day 7 post-inoculation. The full workflow implemented in this work is presented in **Figure 1**. First, regions of interest (ROIs) in the tomato canopy were extracted, and the spectral data were preprocessed including smoothing, baseline correction, and normalization. Then, CWT was used to enhance weak spectral signals associated with early-stage stress of BW, thereby improving the sensitivity of the spectrum to physiological changes in the initial infection stage. Using the spectral features extracted by wavelet transform as input and early disease grading as output, a CWT-STNet model was constructed. The performance of this model was compared with traditional methods (SVM, RF and CNN). Furthermore, the SHAP method was used to interpret the CWT-STNet model, identifying key spectral features crucial for early detection of Bacterial Wilt, and subsequently analyzing the spectral response patterns at different stages of disease development.

### 2.1 Experimental design and data collection

#### 2.1.1 Inoculation experiment of tomato bacterial wilt

This study was conducted with three independent experimental batches to ensure the robustness and reproducibility of the data. In each batch, *Ailsa Craig (AC)* was used as the experimental material. Uniform and well-developed seeds of *AC* variety were selected and surface disinfection was performed via immersion in warm water at 55 °C for 15 minutes and subsequently placed into sterile Petri dishes with moist filter paper. These plates were cultured in darkness at 30 °C to promote germination. After germination, uniformly developed seedlings were then transplanted into plastic pots with a top diameter of 10 cm and a height of 9 cm, and only one seedling was grown in each pot. Each pot contained 0.5 kg of commercially available seedling substrate sterilized by autoclaving. All plants were grown in an artificial climate chamber under controlled conditions: temperature of 25 ± 1 °C, 16 h light/8 h dark photoperiod, light intensity of 2000 lux, and relative humidity of 60–70%.

**Figure 2:**
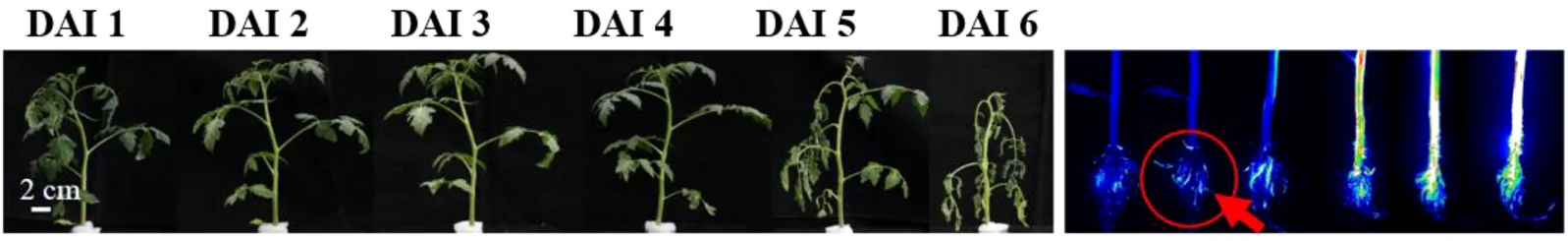
Representative tomato plants and corresponding bioluminescence images used to confirm *Ralstonia solanacearum* colonization following inoculation with the lux-tagged strain FJ91-LUX. The left panel shows representative tomato plants at 1–6 days after inoculation (DAI), illustrating the progression of bacterial wilt symptoms over time. The right panel shows the corresponding bioluminescence images of the same plants. Bioluminescent signals were detectable as early as 1–2 DAI before obvious wilting symptoms became visible (red circle), confirming successful bacterial colonization prior to symptom expression. Signal intensity increased with disease progression, consistent with pathogen multiplication in planta. These results confirm that infection status was verified by bioluminescence imaging rather than inoculation treatment alone.

Following a 30-day cultivation period after transplantation, the experiment was conducted in three independent batches. Each batch consisted of 75 healthy tomato plants with uniform growth and no visible pests or diseases. Within each batch, 65 plants were assigned to the infection group and inoculated using the root-drenching method, while the remaining 10 plants served as the healthy control group and received 20 mL of sterile distilled water using the same procedure. Hyperspectral data were collected from 1 to 6 days after inoculation (DAI) in each batch. All three batches were conducted under identical controlled environmental conditions using the same artificial climate chamber, inoculation procedure, hyperspectral imaging system, acquisition protocol, and environmental settings. Therefore, the data from the three batches were pooled for subsequent analyses. The bacterial strain used was *FJ91-LUX* (Xu et al., 2022). Successful infection of inoculated plants was confirmed by bioluminescence imaging using the lux-labelled strain FJ91-LUX. Bioluminescence signals were detected in inoculated plants before visible wilting symptoms developed, confirming bacterial colonization and providing reliable disease labels for subsequent model development and evaluation (**Figure 2**). After inoculation, all plants were returned to the climate chamber and maintained under the same environmental conditions as before treatment. The day of inoculation was defined as 0 days after inoculation (DAI). Disease symptoms were recorded daily at 13:00, and canopy spectral data were simultaneously acquired using a hyperspectral imaging system.

#### 2.1.2 Determination of disease severity levels and identification of critical early stages for modeling

**Figure 3:**
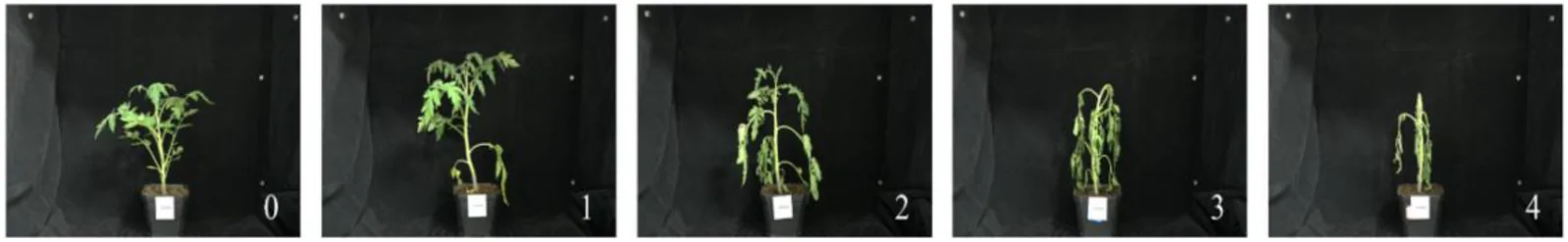
Symptom progression of tomato bacterial wilt. Representative plants showing foliar wilting symptoms corresponding to disease severity levels 0 (healthy control) to 4 (severe wilting).

The severity of bacterial wilt was scored by expert visual assessment of the percentage of tomato wilting leaves, following the criteria described (Wang et al., 2023b). Disease severity was graded into five levels (**Figure 3**): Level 0, (healthy asymptomatic control); Level 1, (infected with either no visible wilting symptoms or 1%-25% of leaves wilted); Level 2, (infected and 26%-50% of leaves wilted); Level 3, (infected and 51%-75% of leaves wilted); Level 4, (infected and 75%-100% of leaves wilted). Notably, the levels 1 and 2 were considered the early infection stage, representing the asymptomatic and mildly symptomatic phases used for model development and evaluation.

#### 2.1.3 Hyperspectral imaging platform and data acquisition

**Figure 4:**
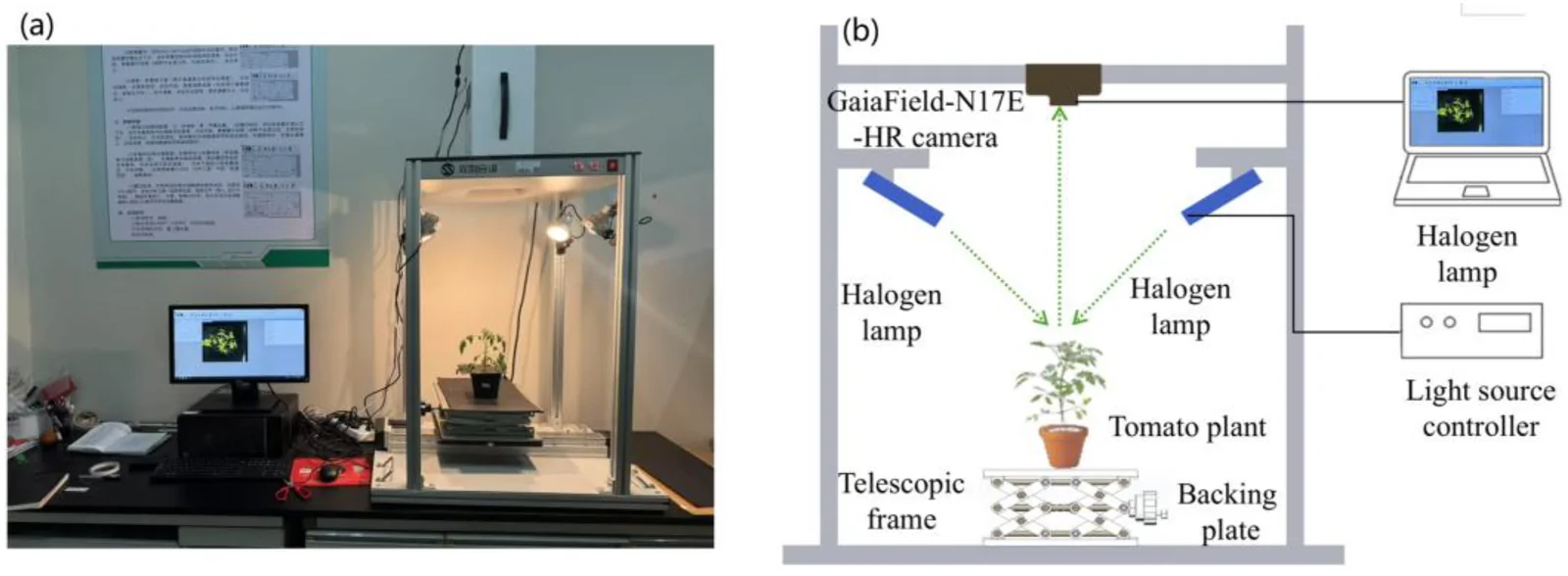
Hyperspectral imaging and data acquisition platform used in this study. **(a)** Photograph of the hyperspectral imaging platform; **(b)** schematic diagram of the hyperspectral imaging system.

The hyperspectral imaging platform included a Dualix GaiaField-N17E-HR Spectral Camera (Dualix Spectral Imaging Technology Co., Ltd., Chengdu, China), four 150 W halogen lamps symmetrically positioned at an illumination angle of 45° relative to the sample, an illumination source controller, and a PC equipped with SpectraVIEW (**Figure 4**). The GaiaField-N17E-HR camera operated in push-broom scanning mode, that enabled hyperspectral image acquisition without moving the samples or the camera during measurement. The spatial resolution of the hyperspectral images was 640 lines × 700 pixels per line. A total of 512 bands were recorded within the spectral range of 850–1700 nm. This scanning speed was set to 8s per hyperspectral cube, while the illumination source intensity was held steady during image acquisition. To eliminate the effect of differences in tomato plant height, a telescopic stand was placed under each flowerpot to maintain a constant spacing between the camera lens and the specimen. The camera-to-sample distance was maintained at 40 cm using a telescopic stand, and the imaging field of view was adjusted to fully cover the tomato canopy during each scan. In addition, a black-white calibration procedure (Equation 1) was performed for each scan to convert the raw hyperspectral images into reflectance data.

(1) $$R=\frac{R_{0}-D}{W-D}\times 100$$

where R denotes the reflectance value of the image after black-and-white correction, *R*_0_ represents the raw sample hyperspectral image, D is the dark standard image, and W refers to the white standard image.

### 2.2 Hyperspectral image pre-processing

#### 2.2.1 Acquisition of region of interest and spectral preprocessing

**Figure 5:**
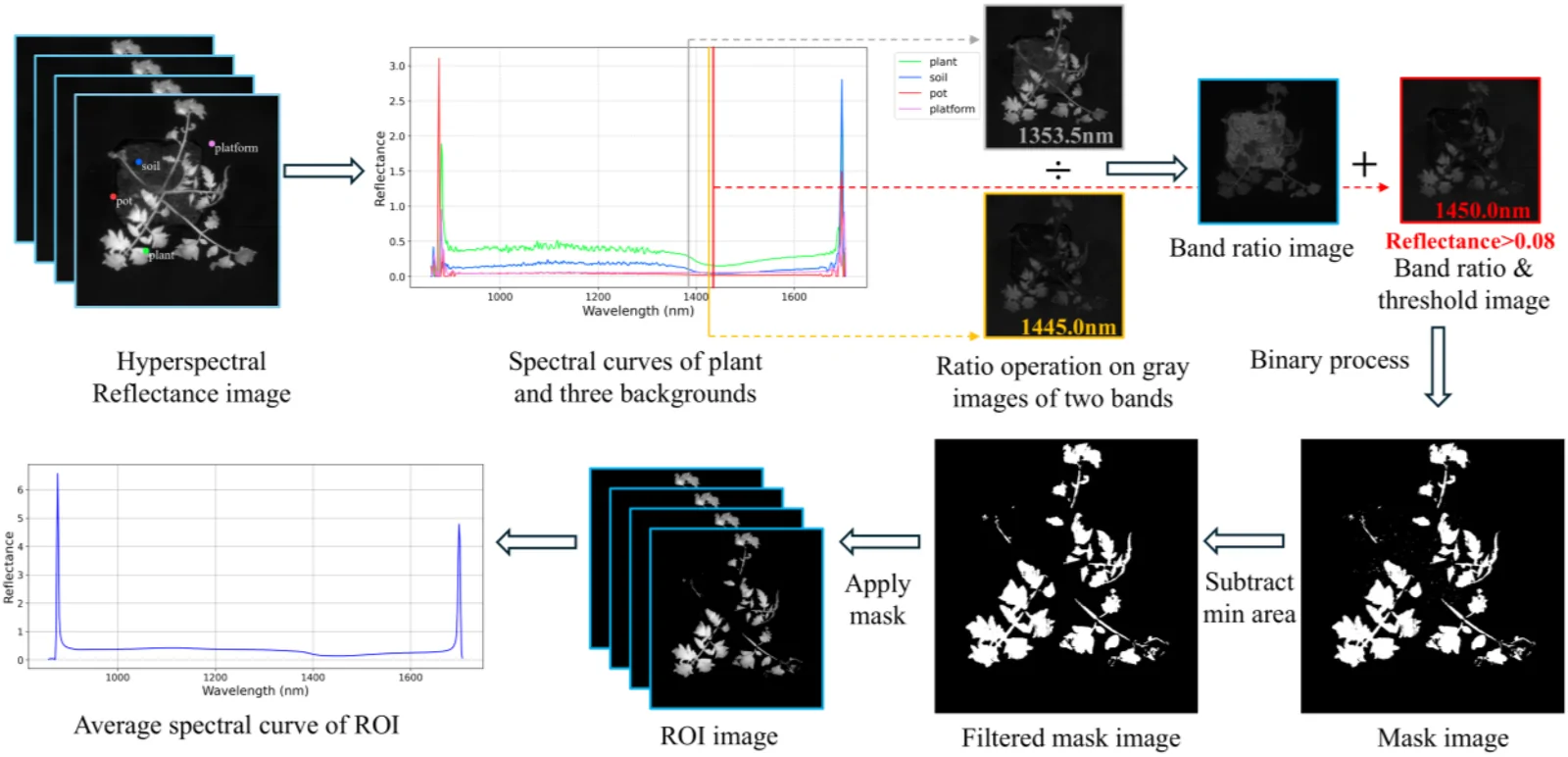
Technical flowchart for extracting regions of interest (ROI) and obtaining average canopy spectra from hyperspectral images of infected tomatoes.

To isolate plant pixels from the background, the leaves of tomato canopies were selected as ROI. ROI extraction and spectrum acquisition were performed based on differences in spectral characteristics between tomato plants and background (flowerpot, soil, and platform; **Figure 5**). By comparing spectral curves, the reflectance of tomato canopies at 1353.5 nm was greatly higher than that at 1445.0 nm, whereas this characteristic was not observed in the background materials. Furthermore, the reflectance of tomato plants at 1450.0 nm was notably higher than that of the three background components. Based on these characteristics, a band-ratio image was generated from the grayscale images at 1353.5 nm and 1445.0 nm. An additional constraint—reflectance at 1450.0 nm greater than 0.08—was applied to improve segmentation accuracy. The ratio image and the threshold image were then combined to generate an initial mask image using threshold segmentation. After removing small noise regions, the ROI was finally extracted by the refined mask image multiplied with the hyperspectral reflectance image. The raw spectral data of each tomato sample after infection was obtained by averaging the spectral reflectance of all pixels within the acquired ROI. Finally, a series of preprocessing operations were implemented on the raw spectral data, including baseline correction (Peng et al., 2026), Savitzky-Golay smoothing for noise reduction (Savitzky and Golay, 1964), multiplicative scatter correction, min-max normalization, first derivative transformation (Wang et al., 2026), and deconvolution based on the Wiener filter.

#### 2.2.2 Continuous wavelet transform

CWT is a multiscale signal analysis tool that transforms one-dimensional spectral signals into two-dimensional wavelength-scale representations, enabling the simultaneous capture of fine-scale high-frequency details and broad low-frequency trends (Cheng et al., 2010). In this study, CWT was utilized to process the average raw reflectance spectrum of the extracted tomato canopy region of interest. The narrow absorption band and wide reflectance variation range of the ROI diseased area can be preserved by converting the one-dimensional spectrum into a two-dimensional wavelet coefficient space, thereby enhancing the subtle spectral features associated with early bacterial wilt stress. Specifically, the Mexican Hat Wavelet was selected as the mother wavelet due to its superior frequency-scale localization, which facilitates the resolution of fine spectral structures of ROI diseased area. Wavelet coefficients were computed by calculating the inner product of the wavelet function at multiple scales with the average raw reflectance spectrum. The low scale (1-5) coefficients captured the high-frequency details corresponding to acute absorption features of ROI diseased area, which is critical for detecting early-stage physiological and biochemical changes caused by BW stress. High-scale coefficients reflect low-frequency background trends and were largely discarded in order to reduce redundancy. All CWT computations were performed using the wavelet toolbox in IDL 8.3.

### 2.3 Proposed early-stage disease detection model of CWT-STNet

**Figure 6:**
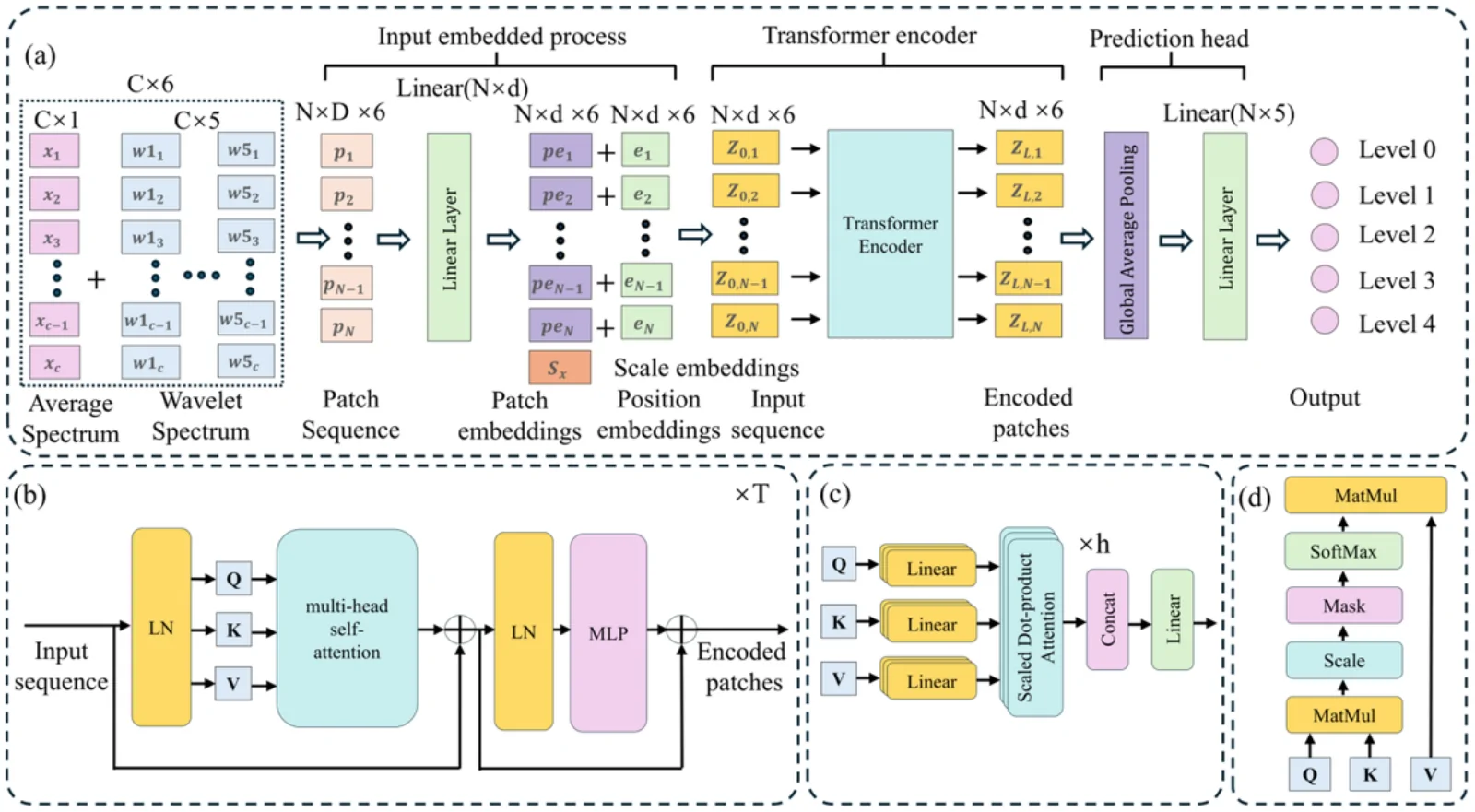
Framework of the proposed CWT-STNet model for early-stage detection of tomato bacterial wilt including its internal sub-modules. **(a)** The overall structure of the CWT-STNet model; **(b)** transformer encoder; **(c)** multi-head self-attention mechanism; **(d)** scaled dot-product self-attention.

An early-stage disease detection model based on Continuous Wavelet Transform and a Spectral Transformer Network (namely, CWT-STNet) was developed in this study, and its overall framework is shown in **Figure 6**. The CWT-STNet model was comprised of three core modules: an input embedding layer, a Transformer encoder, and a prediction head.

#### 2.3.1 Decomposition of the CWT-STNet: embedding, encoding, and identification

First, hyperspectral reflectance data were transformed into a time–frequency representation using CWT. This transformation enabled the decomposition of spectral signals into multi-scale components within a wavelength–scale two-dimensional space, thereby enhancing subtle spectral features associated with early disease stress. The CWT-derived feature maps and spectral features were coupled as inputs and fed into the input embedding layer (**Figure 6a**). In this layer, the two-dimensional spectral representations were reshaped into a sequence of feature tokens. Linear projection was applied to project the preprocessed ROI diseased areas as input features into a high-dimensional embedding space. Positional and scale encoding was further introduced to preserve the sequential order and relative relationships among spectral bands of ROI diseased area.

Subsequently, the embedded feature sequence was processed by the Transformer encoder (**Figure 6b**). The encoder consisted of multiple stacked layers, each including a multilayer perceptron and a multi-head self-attention mechanism (**Figure 6c**). The self-attention mechanism (**Figure 6d**) enabled the model to capture global contextual relationships within spectral bands of ROI diseased area, allowing long-range interactions and subtle variations in spectral responses to be effectively modeled. Meanwhile, the feed-forward network enhanced the nonlinear representation capability of the Transformer model. Finally, the output features from the Transformer encoder were passed to the prediction head for identification. The prediction head consisted of dense layers with a final Softmax function (Bridle, 1990), yielding the likelihood distribution of disease severity.

#### 2.3.2 Training strategy and model configuration of CWT-STNet

To enhance the generalization ability and early-stage disease detection accuracy of CWT-STNet, a two-stage training strategy was adopted, consisting of self-supervised pre-training based on a masked autoencoder framework followed by supervised fine-tuning. During the pre-training stage, 75% of the input embeddings are randomly masked, and the complete spectrum was reconstructed from the remaining unmasked tokens. This strategy encouraged the encoder to learn robust disease-related feature representations. In the subsequent fine-tuning stage, the pre-trained encoder weights were applied to the backbone of the CWT-STNet model. To address the imbalance in severity grading of bacterial wilt, a weighted cross-entropy loss function (Wang et al., 2023b) was then employed in the model.

Furthermore, the key hyperparameters of the CWT-STNet model were systematically configured and optimized. A random search strategy with 20 iterations was employed, using the Kappa coefficient as the optimization criterion. The search space covered several important parameters, including learning rate decay, batch size, dropout rate, weight decay, number of attention heads, model dimension, feed-forward network dimension, data augmentation probability, and noise standard deviation.

#### 2.3.3 Model interpretability analysis and disease severity grading mapping

This study employed the SHAP method to improve the interpretability of the CWT-STNet model, quantify the contribution of multi-scale wavelet spectral features to the early-stage detection of BW, and analyze the spectral response patterns at different stages of disease development. We used five-fold cross-validation to train and evaluate the CWT-STNet model. The deep SHAP algorithm was used to calculate the SHAP feature values ​​of the trained CWT-STNet model at different wavelet scales of the ROI diseased areas (Xu et al., 2024). To improve model robustness, the average absolute SHAP values ​​of each fold were aggregated and averaged to assess feature importance, and the top 10 most influential key features were selected. These features correspond to specific wavelet scales and wavelength regions and are closely related to the key physiological responses of tomato plants to bacterial wilt infection.

Subsequently, the trained CWT-STNet model was then used to generate distribution maps of tomato canopy leaf lesions at different disease severity levels. The corresponding wavelengths were extracted as target spectral regions, and a threshold was determined using the ROC-Youden index (Youden, 1950). Based on this threshold, binary masks were generated to identify the regions that show strong disease-related spectral responses. These regions were then visualized by superimposing a fluorescence-like highlighting effect on the grayscale hyperspectral image, so as to visually present the spatial distribution and severity of bacterial wilt infection.

### 2.4 Comparative models

To comprehensively evaluate the performance of the proposed CWT-STNet model, we compared it with widely used benchmark models including machine learning models (RF, SVM) as well as deep learning models (CNN). These models represent the classic methods and the most cutting-edge methods in spectral data analysis, which established a reference standard for assessing the performance of our model. All models used two types of input data: five CWT multi-scale spectral features and Original spectral features and were trained and evaluated through a unified experimental process to ensure the comparability of the results.

#### 2.4.1 Traditional machine learning models

SVM can achieve interval margin identification between different categories by finding the optimal hyperplane. It is suitable for identification tasks in high-dimensional feature spaces and show stable performance and strong generalization ability on small data sets. The default kernel function was set to the Radial Basis Function, and hyperparameters including regularization parameter C, kernel-related parameters (γ and function type), and polynomial kernel degree were optimized via 2-fold cross-validation combined with random search (Nagasubramanian et al., 2018).

RF was based on ensemble learning and performs identification by building multiple decision trees and integrating voting mechanisms. This method can effectively handle high-dimensional data and complex nonlinear relationships, besides having strong resistance to overfitting (Mahlein et al., 2018). A total of 100 decision trees was fixed, and parameters such as the number of decision trees, maximum tree depth, minimum number of samples required for internal node splitting, and splitting criteria were optimized in this study. Both models were trained separately on multi-scale spectra and single original spectrum as competitive models for performance evaluation.

#### 2.4.2 Deep learning model

CNN can capture local features of spectral data through 1D convolutional layers, reduce dimensionality with pooling layers, then achieve identification by fully connected layers, enabling automatic learning of hierarchical features from spectral data (Vásconez et al., 2024). The model was trained separately on multi-scale spectra and single original spectrum: when using multi-scale spectra, each wavelet-scale branch comprises three 1D convolutional blocks with respective kernel sizes of 7, 5, and 3, and output channels of 32, 64, and 128, respectively; batch normalization, ReLU activation, max-pooling (pooling kernel size of 2), and dropout layers were sequentially connected after the convolutional layers; multi-scale features were fused through a 256-dimensional fully connected layer and then fed into the identification head to output class probabilities. When using a single original spectrum, the model structure remained consistent, with only the input being original spectral data.

ResNet18 was additionally employed as a representative deep convolutional network for comparison with the proposed Transformer-based architecture. The standard ResNet18 architecture was adapted for one-dimensional spectral data by replacing the original 2D convolutional layers with 1D convolutions, while retaining the residual learning framework and four sequential residual stages. The model was trained separately using the original spectra and CWT-transformed multi-scale spectra under the same training and evaluation settings as the other deep learning models. For the CWT-ResNet18 model, the multi-scale spectral features were provided as the network input, whereas the ResNet18 model used the original single spectrum. The final fully connected layer was modified to match the number of bacterial wilt severity classes and output the corresponding class probabilities.

All models were optimized for optimal hyperparameter combinations via 2-fold cross-validation combined with random search, with the Kappa coefficient as the optimization objective. A normalization layer and Gaussian noise augmentation (standard deviation of 0.01, augmentation probability of 0.5) were introduced during the training phase to enhance generalization ability. Finally, model performance was evaluated through five-fold cross-validation, with the mean and standard deviation of identification metrics used as the final evaluation results. Both CNN and CWT-STNet models adopted a two-stage training paradigm.

### 2.5 Model evaluation

The model’s overall effectiveness is evaluated using five indicators as follows: average precision (Equation 2), average recall (Equation 3), average F1-score (Equation 4), overall accuracy (Equation 5), and kappa coefficient (Equation 6). Average precision, average recall, F1 score, and overall accuracy all range from 0 to 1. Generally, the closer these indicators are to 1, the better the model is at capturing true positive samples and reducing false negatives (Zhang et al., 2021). A value greater than 0.8 is often considered indicative of good model performance. The Kappa coefficient ranges from -1 to 1, and a value greater than 0.8 indicates that the model’s predictions agree almost perfectly with the true labels (Foody, 2020).

(2) $$Average\;Precision=\frac{1}{c}\sum_{i=1}^{c}\frac{TP_{i}}{TP_{i}+FP_{i}}\;$$

(3) $$Average\;Recall=\frac{1}{c}\sum_{i=1}^{c}\frac{TP_{i}}{TP_{i}+FN_{i}}$$

(4) $$Average\;F1=\frac{1}{c}\sum_{i=1}^{c}\frac{2\times {\text{Precision}}_{i}\times {\text{Recall}}_{i}}{{\text{Precision}}_{i}+{\text{Recall}}_{i}}$$

where *TP_i_* denotes the count of samples that are accurately classified into grade *i*, *FP_i_* represents the quantity of samples incorrectly assigned to grade *i*, *TN_i_* is the count of samples accurately recognized as belonging to any grade other than *i*, and *FN_i_* stands for the total samples in grade *i* that are misclassified into other grades.

(5) $$Overall\;accuracy=\frac{\sum_{i=1}^{c}TP_{i}}{N}$$

with N being the count of test samples.

(6) $$Kappa\;coefficient=\frac{p_{0}-p_{e}}{1-p_{e}}$$

where *p*_0_ stands for the total classification accuracy, and *p_e_* can be derived as follows (Equation 7):

(7) $$p_{e}=\frac{\sum_{i=1}^{c}a_{i}\times b_{i}}{N^{2}}$$

where *a_i_* denotes the actual quantity of samples belonging to grade *i*, and *b_i_* represents the count of samples classified as grade *i* by the model.

## 3 Results

### 3.1 Spectral curve analysis of tomato bacterial wilt

**Figure 7:**
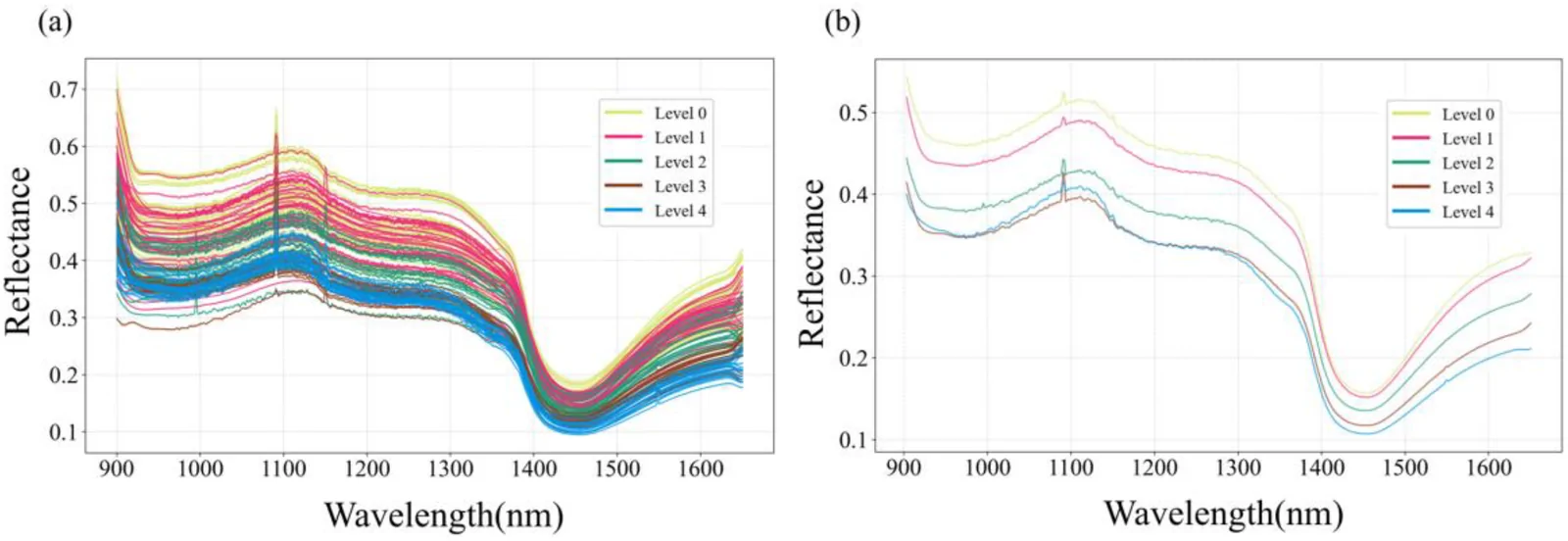
Spectral reflectance curves of tomato canopies under different bacterial wilt (BW) severity levels. **(a)** Raw spectral reflectance of all sampled canopy pixels (900–1650 nm); **(b)** Averaged spectral reflectance curves per severity level (900–1650 nm).

The spectral reflectance characteristics of tomato leaves decreased progressively with increasing wavelength (**Figure 7**). At 900 nm, the reflectance was about 0.64 across all severity levels (Level 0 to Level 4). As wavelength increased to 1600 nm, the value of reflectance gradually declined to around 0.40. After infection with *Ralstonia solanacearum*, the spectral reflectance values remained consistent in different disease severity levels of BW at each wavelength (**Figure 7a**). For instance, at 1100 nm, all severity levels of BW exhibited a reflectance of 0.50; at 1300 nm, all showed 0.46; and at 1500 nm, all displayed 0.42. Even at Level 4 (the most severe infection stage of BW), the reflectance did not deviate from other levels. Averaging spectral reflectance within the same disease severity level revealed clearer separations among different disease severity levels (900–1650 nm) (**Figure 7b**). In the near-infrared (NIR, 900–1100 nm) region, healthy plants (Level 0) exhibit the highest reflectance, which gradually decreases with increasing disease severity. Similarly, in the short-wave infrared (SWIR, approximately 1100–1650 nm) region, healthy plants display the highest reflectance, which progressively declines as the disease advances. A pronounced absorption feature is observed around 1400 nm, where severely infected plants (Level 4) showed the lowest reflectance among all groups, indicating strong water-related absorption characteristics. These results indicated that the averaged spectral reflectance of tomato canopies does not exhibit clear differentiation among bacterial wilt severity levels based solely on raw reflectance values. The spectral curves for all severity levels of BW were almost identical, which suggested that the original reflectance characteristics have limited ability to distinguish between early or advanced stages of bacterial wilt infection.

### 3.2 Comparative analysis of original and wavelet-transformed spectra for disease detection

**Figure 8:**
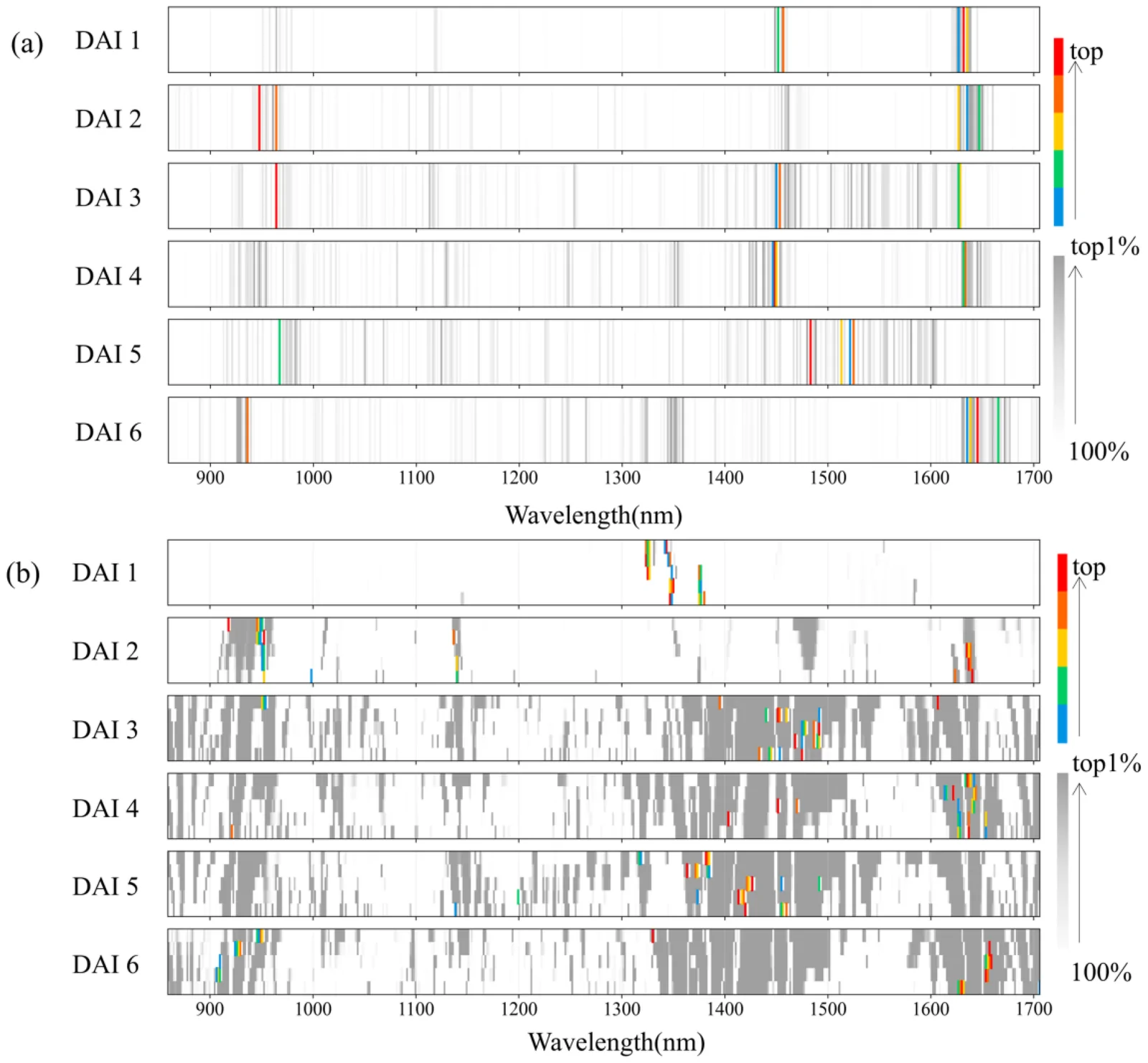
Feature sensitivity of tomato plants for early bacterial wilt detection based on **(a)** original reflectance spectra and **(b)** continuous wavelet transform (CWT)-derived features. Grayscale intensity indicates the sensitivity of each feature, with higher brightness corresponding to greater sensitivity, while colored patches denote the top 1% most sensitive features for disease discrimination.

To elucidate the spectral response patterns of tomato plants under bacterial wilt infection, hyperspectral data collected from 1 to 6 days after inoculation (DAI) were analyzed (**Figure 8**). The original reflectance spectrum exhibited moderate sensitivity across most wavelengths and DAI. Sensitivity gradually improved over time, with stable performance observed starting at DAI 3 in several key wavelength regions (e.g., 950–980 nm and 1400–1500 nm), where near 100% sensitivity was achieved. The top 1% most sensitive bands of the original reflectance spectrum were primarily distributed in the NIR (850–1100 nm) and SWIR (1400–1700 nm) regions throughout DAI 1-6 (**Figure 8a**). The sensitive feature regions remained relatively stable over time, with NIR features mainly concentrated around 900–1000 nm and SWIR features around 1400–1500 nm and 1600–1700 nm. However, the overall distribution of high-sensitivity features is sparse at early stages and gradually expands as the disease progresses, indicating that the spectral response to infection becomes increasingly pronounced over time.

In contrast, the reflectance spectrum after CWT-transformed substantially enhanced early-stage sensitivity (**Figure 8b**). At day 1 post-inoculation, only a few isolated high-sensitivity features were observed, primarily around 1300–1400 nm, suggesting that the disease signal was still weak and localized. As the infection develops (DAI 2-6), the spatial coverage of informative features expands substantially, eventually spanning nearly the entire 900–1700 nm range by DAI 3-6. The top 1% most sensitive features (colored patches) are predominantly distributed in the CWT-transformed domain, particularly across DAI 3 to DAI 6, covering a broader and more continuous wavelength range compared to the original spectra. This indicated that CWT effectively enhanced weak spectral signals and enabled the extraction of multi-scale features that are not detectable in the original spectra. Notably, wavelet analysis identified not only the key high-importance features captured by original spectra but also a large number of basic features that remain undetected in the original analysis. This more comprehensive representation of spectral dynamics under pathogen infection underscores the advantage of CWT-based feature extraction for early disease detection.

### 3.3 Identification results of tomato bacterial wilt

#### 3.3.1 Performance comparison of the CWT-STNet model and the traditional models

The performance of the proposed CWT-STNet model and the traditional models (i.e., RF, SVM, CNN and ResNet18) for detecting the early-stages of tomato infection is summarized in **Table 1**. The reported values represent the mean ± standard deviation of the evaluation metrics obtained from 5-fold cross-validation. Across all model types, the incorporation of CWT consistently improved detection performance, as evidenced by the superior results of CWT-SVM, CWT-RF, CWT-CNN, CWT-ResNet18, and CWT-STNet compared with their counterparts based on raw spectra. This indicated that CWT effectively enhances discriminative spectral features and reduces noise and redundancy in hyperspectral data. Among the deep learning models trained on raw spectra, CNN and STNet outperformed traditional machine learning approaches (SVM and RF), whereas ResNet18 showed comparatively lower performance. Within the traditional methods, SVM (average precision: 77.35%) showed slightly better performance than RF (average precision: 75.62%). After CWT processing, SVM and RF exhibited substantial improvements across all evaluation metrics, suggesting that CWT particularly benefits models that are more responsive to feature quality. However, both SVM and RF exhibited relatively large variations across the five folds, whereas the deep learning models showed substantially more stable performance, indicating better robustness to variations in the training data.

**Table 1:** Comparison of the proposed CWT-STNet model with traditional models (RF, SVM, CNN, ResNet18) in identifying early stages of bacterial wilt in tomatoes.

Model name	Precision (%)	Recall (%)	F1 (%)	OA (%)	Kappa
SVM	77.35 ± 27.75	76.68 ± 28.62	76.98 ± 28.20	74.53 ± 31.39	0.68 ± 0.38
CWT-SVM	85.93 ± 6.15	87.09 ± 5.10	86.50 ± 5.62	90.28 ± 6.29	0.86 ± 0.05
RF	75.62 ± 29.09	73.07 ± 31.83	74.14 ± 30.62	76.24 ± 28.41	0.67 ± 0.39
CWT-RF	81.49 ± 6.97	84.07 ± 6.20	82.72 ± 6.42	83.81 ± 7.08	0.78 ± 0.09
CNN	86.12 ± 2.66	85.18 ± 1.87	85.64 ± 2.13	91.31 ± 1.46	0.88 ± 0.02
CWT-CNN	89.10 ± 2.47	88.66 ± 2.48	88.88 ± 2.46	93.01 ± 1.84	0.90 ± 0.02
STNet	85.76 ± 4.12	84.89 ± 4.44	85.32 ± 4.26	89.95 ± 2.73	0.86 ± 0.03
CWT- STNet	95.03 ± 2.38	94.60 ± 2.98	94.81 ± 2.59	96.77 ± 1.66	0.95 ± 0.02
ResNet18	70.72 ± 7.49	62.66 ± 4.18	66.31 ± 5.08	72.90 ± 4.04	0.62 ± 0.05
CWT- ResNet18	89.91 ± 6.19	89.23 ± 5.41	89.56 ± 5.75	91.66 ± 4.61	0.88 ± 0.06

For deep learning models based on raw spectra, CNN achieves slightly better performance than STNet, with an average precision of 86.12%, overall accuracy of 91.31%, and Kappa coefficient of 0.88, compared to 85.76%, 89.95%, and 86.31 for STNet, respectively. ResNet18 was also included as a conventional convolutional neural network baseline and showed lower performance on the raw spectra. After CWT transformation, CWT-ResNet18 achieved an overall accuracy (OA) of 91.66%, representing an improvement of 18.76 percentage points compared with the original ResNet18. After integrating CWT, all models showed consistent and notable improvements, demonstrating that multi-scale spectral feature extraction further enhances the representation learning capability of deep networks. Notably, the proposed CWT-STNet model attained the top overall performance across all models, yielding the highest values across all evaluation metrics, including an average precision of 95.03%, average recall of 94.60%, average F1-score of 94.81%, overall accuracy of 96.77%, and Kappa coefficient of 0.95. CWT-CNN achieved the second-highest OA (93.01%), with slightly lower but comparable performance. Compared with CWT-ResNet18, CWT-STNet improved OA by 5.11 percentage points, further highlighting the advantage of the proposed architecture in extracting disease-relevant spectral information. These results demonstrated that the integration of CWT-based multi-scale feature enhancement with Transformer-based representation learning enables more comprehensive extraction of disease-relevant spectral information, thereby further improving identification accuracy. The CWT-STNet model further improved overall accuracy by 6.82 percentage points compared with the original STNet model, demonstrating that CWT transformation coupled with a dedicated network architecture provides a highly effective strategy for early bacterial wilt diagnosis. Overall, the results in **Table 1** demonstrate that CWT consistently enhances the performance of both conventional machine learning and deep learning models, while the combination of CWT with the Transformer-based STNet architecture achieved the most favorable performance among all evaluated models.

#### 3.3.2 Superior performance of the proposed CWT-STNet in early-stage BW detection

**Figure 9:**
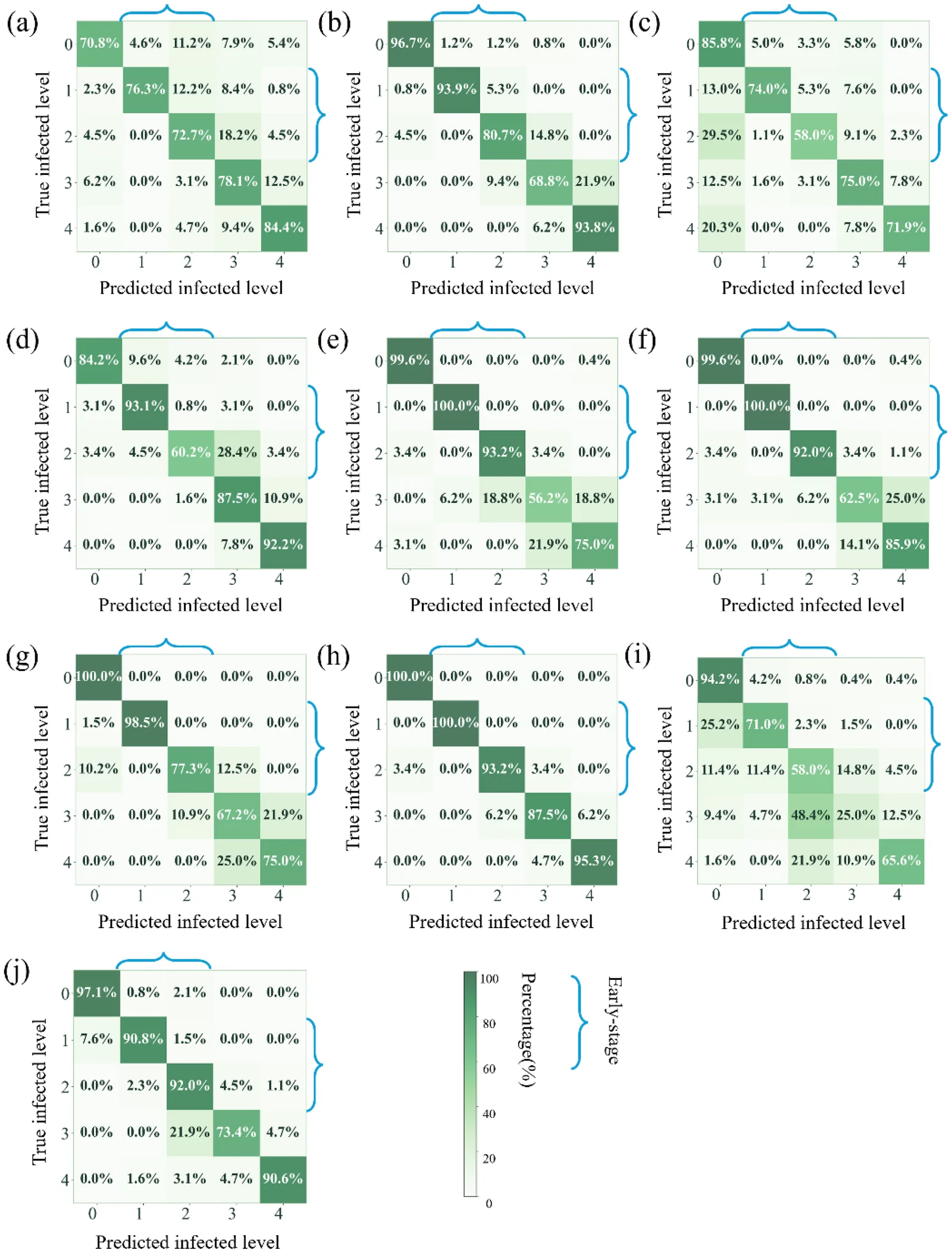
Confusion matrices for tomato bacterial wilt severity identification. **(a–d)** Traditional machine learning models; **(e-j)** deep learning models. Early-stage disease levels (1 and 2) are highlighted to compare detection sensitivity across models. **(a)** SVM, **(b)** CWT-SVM, **(c)** RF, **(d)** CWT-RF, **(e)** CNN, **(f)** CWT-CNN, **(g)** STNet, **(h)** CWT-STNet, **(i)** ResNet18, **(j)** CWT- ResNet18.

Confusion matrices were applied to quantify the identification performance for different models across disease severity levels, with a particular focus on the diagnosis of early-stage samples (Levels 1 and 2) (**Figure 9**). Across all model types, the incorporation of CWT led to consistent improvements in identification accuracy at each severity level. For example, the accuracies for early-stage samples (Levels 1 and 2) were increased after CWT processing for SVM, RF, CNN and STNet, with improvements of (ΔLevel 1: 87.2%→96.8%) and (ΔLevel 2: 75.3%→81.5%), respectively, indicating enhanced discrimination of subtle spectral differences. A similar improvement was also observed for ResNet18 after CWT processing, with the classification accuracy for Level 1 increasing from 71.0% to 90.8% and that for Level 2 increasing from 58.0% to 92.0%. Among all models, the proposed CWT-STNet model essentially achieved the highest identification accuracy across all severity levels. Specifically, it reaches 100% accuracy for healthy samples (Level 0), 100% for Level 1, and 93.2% for Level 2 (**Figure 9h**). In comparison, the second-best model, CWT-CNN, had accuracies of (Level 1: 100%) and (Level 2:92%) (**Figure 9f**); however, while its correct classification performance is close, non-negligible misclassifications of other samples as Level 1 and Level 2 still existed, specifically (Level 3→Level 1: 3.1%, Level 3→Level 2: 6.2%), and such errors are somewhat reduced in the CWT-STNet model. In contrast, models based on raw spectra generally exhibit lower accuracies for level early-stage samples (**Figures 9a, c**). Although the deep learning models CNN and STNet have relatively higher accuracies, adjacent severity level samples are more frequently misidentified as early-stage samples, and this difference is further reflected in the confusion patterns; for models using raw spectra, the misidentification rates are (Level 3→Level 1: CNN = 6.2%) and(Level 3→Level 2: CNN = 18.8%, STNet = 10.9%; **Figures 9e, g**). Compared with the original ResNet18 model, CWT-ResNet18 also showed substantially reduced confusion between adjacent disease severity levels, further supporting the effectiveness of CWT in enhancing weak disease-related spectral features (**Figures 9i, j**). In contrast, the CWT-STNet model shows fewer mispredictions of other samples as early-stage samples in the early stage (Level 3→Level 1: 6.2%), and the overall misclassification rate for late-stage samples amounts to merely 14.3%, indicating improved sensitivity to weak disease signals.

#### 3.3.3 Ablation validation of proposed CWT-STNet model

An ablation experiment was performed to evaluate the design rationale for the CWT-STNet model by addressing two key questions: (1) whether the original spectral branch is necessary, and (2) whether multi-scale feature fusion provides advantages over single-scale features. **Table 2** summarized the early-stage identification performance of the CWT-STNet model under different combinations of CWT scales and the inclusion or exclusion of the original spectra. When the original spectra were excluded, the CWT-STNet model achieved an overall accuracy of only 84.15% ± 3.71%, even when all CWT scales were combined. When the original spectral branch was included, the model obtained an overall accuracy of 96.77% ± 1.66%, representing an enhancement of more than 12%. These results indicated that multi-scale CWT features alone could not fully replace the baseline information provided by the original spectra, and that the two feature sources were complementary.

**Table 2:** The proposed CWT-STNet model presents ablation experimental results when using different inputs or combinations of inputs from the original spectrum of the ROI lesion region and CWT transformed spectral data.

CWT scale	Whether the original spectrum included	Overall accuracy (%)
1-5	NO	84.15 ± 3.71
1-5	YES	96.77 ± 1.66
1	YES	94.37 ± 3.08
2	YES	94.20 ± 3.04
3	YES	94.20 ± 3.22
4	YES	94.72 ± 2.39
5	YES	92.67 ± 3.38

When the original spectral branch was retained, the CWT-STNet model achieved overall accuracies ranging from 92.67% to 94.72% using individual CWT scales (1-5). When all CWT scales were combined, the model achieved a higher accuracy of 96.77%, exceeding the performance of all single-scale configurations. These results indicated that different CWT scales captured complementary disease-related information, and that multi-scale fusion improved feature utilization. Overall, the CWT-STNet model achieved the highest identification accuracy when both the original spectral branch and all CWT scales were included. This finding validated the architectural design of the model, in which each component contributed uniquely to performance and their combination was necessary for accurate early-stage identification of tomato bacterial wilt.

### 3.4 Interpretability analysis of the CWT-STNet model and its application in early- diagnosis of bacterial wilt disease

#### 3.4.1 SHAP analysis of the optimal CWT-STNet model

**Figure 10:**
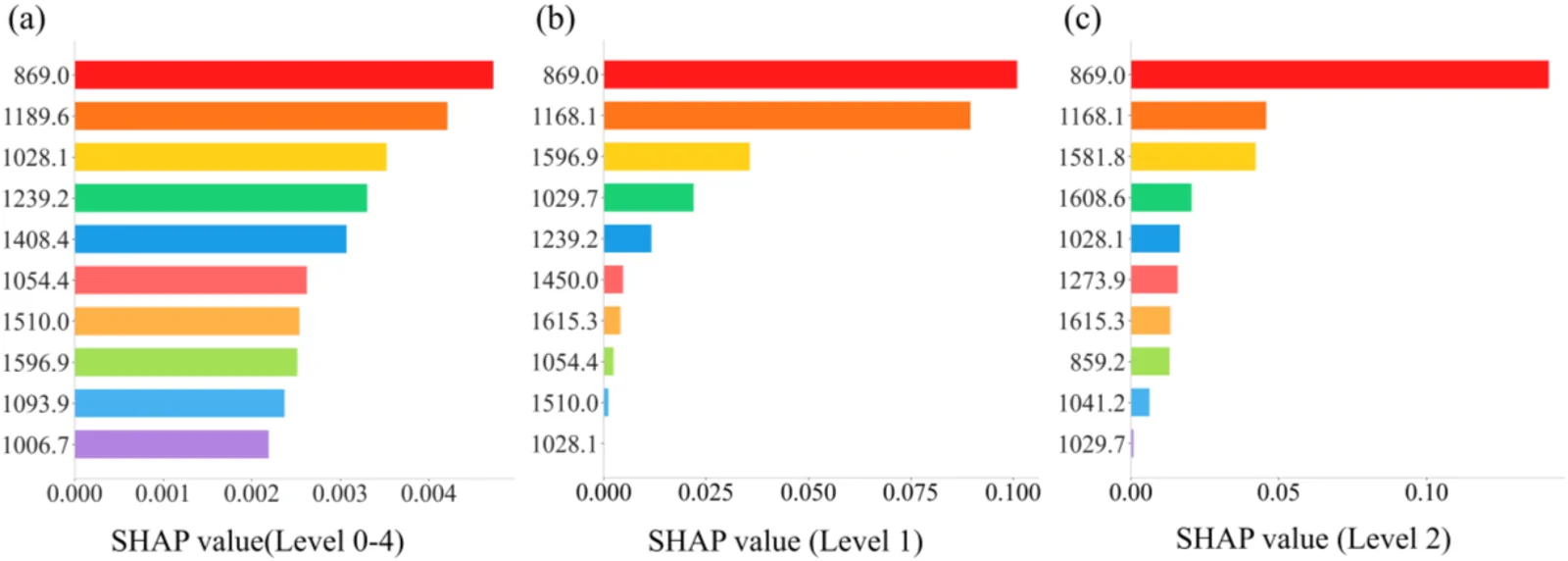
SHAP-based feature importance analysis for early diagnosis of tomato bacterial wilt. **(a)** Global feature importance summary of all samples of tomato bacterial wilt (Level 1, Level 2, Level 3, and Level 4); **(b, c)** were feature importance distribution for early-stage of tomato bacterial wilt (Level 1 and Level 2), respectively. The horizontal axis represents the SHAP value, reflecting the contribution of each feature to model predictions, while the vertical axis lists the key wavelengths (nm) ranked by importance.

To further interpret the decision mechanism of the proposed CWT-STNet model, SHAP analysis was conducted across all disease stages and early infection levels of tomato bacterial wilt (**Figure 10**). In the global analysis of all disease levels (Level 1 to Level 4), the most sensitive wavelengths were around 869.0 nm, 1189.6 nm, and 1028.1 nm, respectively, indicating their strong contributions to model predictions (**Figure 10a**). For the early stage (Level 1) of the disease, the most sensitive wavelengths were 869.0 nm, 1159.6 nm, and 1029.7 nm, with SHAP values of about 0.10, 0.09, and 0.03, respectively, suggesting that these spectral features are critical for distinguishing early infection (**Figure 10b**). For Level 2, the most influential wavelengths were 869.0 nm (SHAP ~0.14), 1168.1 nm (SHAP ~0.046), and 1028.1 nm (SHAP ~0.042), with slight variations in SHAP values compared to Level 1, reflecting differences in spectral responses as the disease progresses. These results demonstrated that specific near-infrared or visible wavelengths played a dominant role in the early detection of tomato bacterial wilt.

#### 3.4.2 Pixel-level infection probability maps generated by the trained CWT-STNet

**Figure 11:**
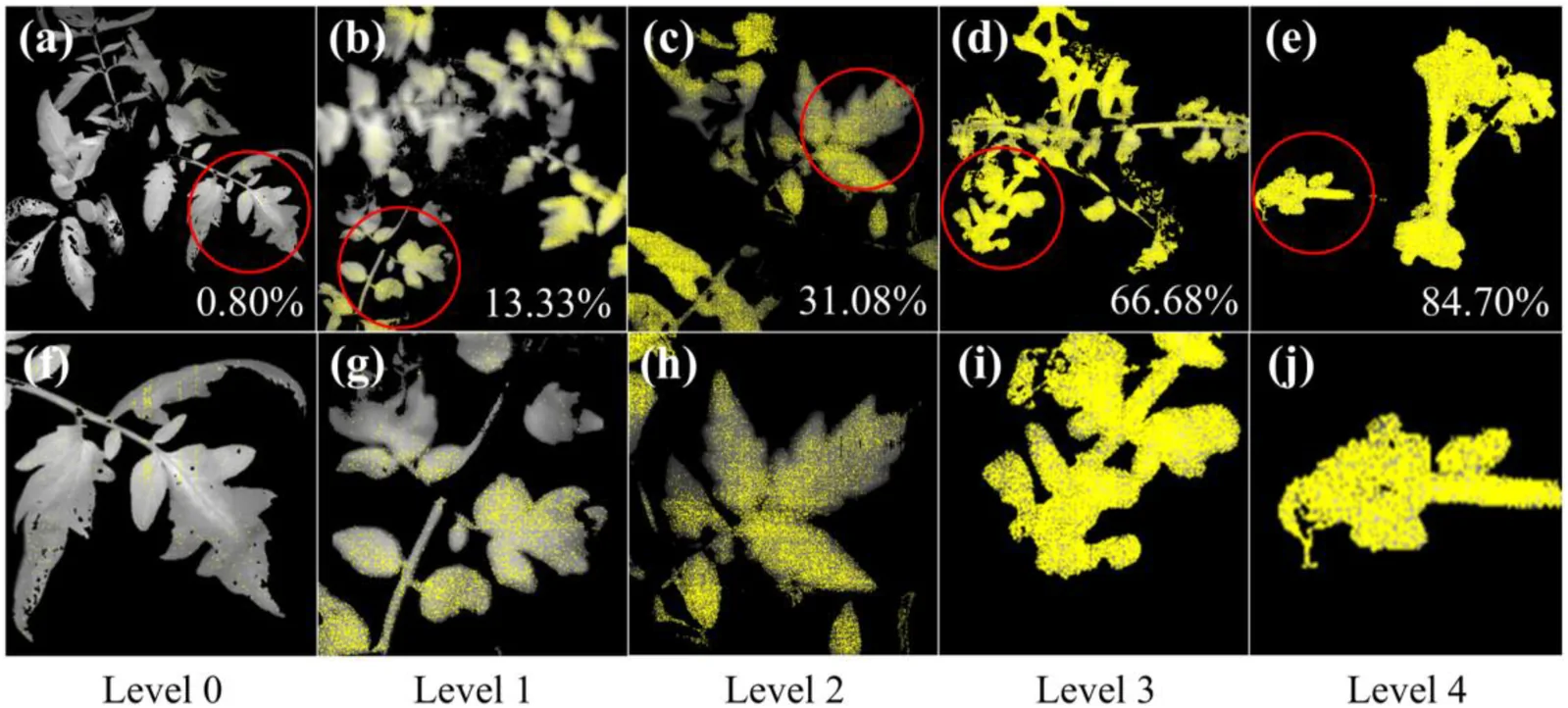
Diagnostic results of the CWT-STNet model for identifying bacterial wilt lesions on tomato canopy leaves. Using hyperspectral data as input, the trained CWT-STNet model generated pixel-level infection probability maps for tomato canopies under different disease severity levels. **(a–e)** Lesion distributions at the tomato canopy; the percentage in the lower-right corner indicated the proportion of pixels detected as infected relative to the total pixel counts of the tomato effective canopy areas. **(f–j)** Enlarged views of local leaves corresponding to the red-circled regions of **(a–e)**, which highlighted the fine-scale spatial distribution characteristics of lesions at the leaf level.

The pixel-level infection probability distribution of bacterial wilt lesions generated by the trained CWT-STNet model provided quantitative information on plant disease progression (**Figure 11**). As BW becomes more severe, the proportion of infected pixels increases greatly from Level 0 to Level 4, rising from 0.80% to 84.70%. For non-infected plants, only a very small number of abnormal pixels were detected (**Figures 11a, i**). For the early-stages of bacterial wilt infection in tomatoes (i.e., Levels 1 and 2), the CWT-STNet model simulated the trend of infected lesions spreading from localized areas to the leaf margins (**Figures 11b, c, g, h**). For the late stages of bacterial wilt infection in tomatoes (after level 3), plants are characterized by obvious drooping and severe wilting. The CWT-STNet model simulated the further expansion of bacterial wilt lesions from the leaves to the whole canopy (**Figures 11d, e, i, j**). These results indicated that the CWT-STNet model cannot only reflect the quantitative changes in disease severity (the proportion of infected pixels increased from 0.80% to 84.70%) but also capture the spatial expansion pattern of lesions from the leaf margin to the entire crown, showing good performance in the refined assessment of plant diseases.

## 4 Discussion

### 4.1 Physiological and spectral basis of early bacterial wilt detection

The reflectance spectral characteristics of tomato plants show systematic and gradual changes as the severity of bacterial wilt increases. The phenomenon is strongly associated with the physiological and biochemical changes in tomato plants caused by infection with *Ralstonia solanacearum*, thereby providing a solid physiological and optical basis for the early detection of the disease (Mahlein, 2016). As shown in the spectral curve (**Figure 7b**), healthy tomato plants (Level 0) exhibit the highest reflectance in both the NIR and SWIR infrared regions, while the reflectance gradually decreases as the disease severity increases. The decrease in reflectance is mainly related to the destruction of the internal structure of leaves caused by pathogen invasion and the reduction of multiple scattering in mesophyll tissues, which has been widely reported in hyperspectral studies on plant stress (Carter and Knapp, 2001; Zhu et al., 2023). An obvious absorption feature was observed around 1400 nm, which further confirms the close relationship between spectral characteristics and leaf moisture status. The 1400 nm wavelength is a typical moisture absorption band, and the absorption intensity in this region is positively correlated with the moisture content of plant tissues, indicating that the physiological changes caused by bacterial wilt are directly reflected in the spectral signals. The phenomenon can be mechanistically explained as follows: the vascular blockage caused by *Ralstonia solanacearum* restricts water transport, leads to tissue dehydration, and enhances the moisture absorption characteristics in the short-wave infrared region (Antony et al., 2025). It is worth noting that even at the early stages of infection (Levels 1 and 2) before visible symptoms appear, subtle spectral differences from healthy plants can be detected. These early spectral deviations may be related to initial physiological disturbances, such as stomatal regulation, slight water imbalances, and early cell degradation. This confirms that hyperspectral technology can capture pre-symptomatic physiological changes, providing feasible conditions for the early non-destructive detection of bacterial wilt.

### 4.2 Mechanistic role of continuous wavelet transform in enhancing disease-related spectral signatures

CWT can effectively improve the accuracy of early detection of plant diseases. Its core advantage lies in its ability to mine subtle, multi-scale spectral features related to potential physiological changes. By decomposing the original spectrum at different scales, CWT can effectively separate overlapping spectral response signals, and this characteristic is crucial for the extraction of early disease signals (Huang et al., 2021). In the early stage of bacterial wilt infection, only minor disturbances occur in the plant’s water status, cell structure, and biochemical components. The corresponding spectral features are weak and localized, often masked in the original reflectance spectrum and thus difficult to identify. As shown in **Figure 8**, the spectrum decomposed by CWT can not only identify the existing key features in the original spectrum but also reveal a large number of potential features that are difficult to capture with the original analysis. This fully demonstrates its unique advantages in weak signal enhancement and hidden feature extraction, and these weak spectral changes are a direct reflection of the physiological disturbances caused by the early infection of bacterial wilt. Therefore, compared with traditional spectral preprocessing methods, CWT has better positioning ability in extracting characteristic wavelengths and can more accurately characterize disease-related spectral responses (Ong et al., 2025; Huang et al., 2021). Furthermore, the multi-scale decomposition inherent in CWT increases the separability of spectral features across disease severity levels by enhancing contrast between subtle physiological differences. This explains the expanded distribution of high-sensitivity features observed in the CWT-transformed data (**Figure 8**). Its advantages are reflected not only at the level of signal processing technology, but also at the level of in-depth mechanism explanation. CWT can establish a direct correlation between the potential physiological disorders of plants and their spectral characteristics, thereby improving the identifiability of early features.

### 4.3 Advantages of using CWT-STNet identification models

The CWT-STNet model performs excellently in the early detection of bacterial wilt, which stems from the synergistic effect generated by CWT-based feature enhancement and the powerful representation learning capability of the Transformer network. CWT functions critically in input feature enhancement: by converting the original spectrum into multi-scale wavelet features, it effectively amplifies the weak signals related to diseases, reduces noise and redundant information, and can provide more comprehensive spectral information compared to using the original spectrum alone. The performance of all models improved consistently when CWT was incorporated (**Figure 9**), especially for early-stage samples (Level 1 and Level 2), which confirms the above conclusion. The Transformer encoder in CWT-STNet contributes effectively to accurate identification. Unlike image data, hyperspectral signals have strong inter-band correlations and long-range dependencies among different wavelengths, meaning that spectral bands are not independent but highly correlated. The multi-head self-attention mechanism of the Transformer encoder can globally model these dependencies and capture subtle spectral interactions that are crucial for early disease identification (Kumar et al., 2025). In contrast, CNN mainly focuses on local feature extraction and may not fully capture long-range spectral relationships. Therefore, the integration of CWT’s multi-scale feature enhancement and Transformer’s global dependency modeling provides a complementary framework for extracting disease-related information. This synergistic effect explains why CWT-STNet achieves the best performance among all models (average precision 95.03%, overall accuracy 96.77%), especially in early detection, indicating its strong robustness and practical application value (**Table 1**). These findings are consistent with recent studies showing that Transformer-based architectures are effective in hyperspectral analysis, particularly in capturing complex spectral dependencies (Vásconez et al., 2024). Recent studies have demonstrated the potential of hyperspectral imaging for early tomato disease detection. For example, Zhang et al. (2024) employed hyperspectral imaging to detect early tomato bacterial leaf spot, demonstrating the feasibility of hyperspectral technology for identifying disease before obvious symptoms developed. Zhou et al. (2025) further combined spectral preprocessing, feature selection, and an optimized BiLSTM model to detect tomato leaf spot and blight diseases, achieving an overall accuracy of 97.3%. However, both studies acquired hyperspectral data from detached tomato leaves under controlled laboratory conditions, where background interference and canopy structural variability were largely minimized. In contrast, the present study focused on non-destructive canopy-level hyperspectral imaging for the early detection of tomato bacterial wilt. Compared with detached-leaf imaging, canopy-level detection is considerably more challenging because spectral measurements are more susceptible to variations in illumination, leaf orientation, canopy architecture, and background interference. Furthermore, tomato bacterial wilt is a vascular disease in which early infection occurs within the xylem before visible symptoms appear, resulting in much weaker spectral responses than those observed in diseases with visible foliar lesions. Despite these challenges, the proposed CWT-STNet effectively enhanced subtle spectral variations through continuous wavelet transform and captured long-range spectral dependencies using a Transformer architecture, demonstrating its potential for practical early bacterial wilt detection under more realistic conditions.

### 4.4 Model interpretability

Through SHAP analysis, this study identified several key spectral wavelengths that made major contributions to tomato bacterial wilt detection, including 869.0, 1189.6, 1239.2, 1408.4, 1510.0, and 1596.9 nm (**Figure 10a**). These wavelengths were mainly distributed in the near-infrared (NIR) and short-wave infrared (SWIR) regions and were closely associated with the physiological changes occurring during bacterial wilt infection. Among them, 869.0 nm exhibited the highest SHAP value across all disease severity levels. This wavelength is located in the NIR region, where spectral reflectance is primarily determined by the internal leaf structure and multiple scattering within the mesophyll tissue (Jacquemoud et al., 1996). In contrast, the SWIR wavelengths, including 1189.6, 1239.2, 1408.4, 1510.0, and 1596.9 nm, are closely related to leaf water status and biochemical composition. Previous studies have demonstrated that reflectance in the SWIR region is strongly influenced by the absorption characteristics of O–H and C–H bonds, enabling the detection of changes in leaf water content and biochemical constituents such as soluble sugars (Curran, 1989; Cheng et al., 2010; Méline et al., 2023). These findings indicate that the key spectral features identified by the model are closely associated with physiological responses, including changes in leaf internal structure, water status, and biochemical composition during tomato bacterial wilt infection, suggesting that the model captures biologically meaningful information related to disease development.

During the early stages of tomato bacterial wilt detection, 869.0 nm exhibited the highest SHAP value (**Figures 10b, c**). As this wavelength primarily reflects the internal structure of leaves, this result indicates that structural changes in leaf tissues had already produced detectable spectral responses during the early stage of infection. This observation is consistent with the sensitivity of NIR reflectance to changes in leaf internal structure (Jacquemoud et al., 1996), further suggesting that this spectral response is closely associated with structural alterations induced by bacterial colonization and proliferation within the xylem, which impairs water transport during bacterial wilt infection. Meanwhile, several SWIR wavelengths exhibited progressively higher SHAP values, reflecting increasingly pronounced spectral responses associated with leaf water deficit and biochemical metabolic changes as disease progressed. These results indicate that, even during the early stages of bacterial wilt, the model was able to identify subtle spectral variations induced by disease development (Curran, 1989; Cheng et al., 2010). Overall, the physiological changes in leaf internal structure, water status, and biochemical composition were closely associated with their corresponding spectral responses, and the model achieved early detection of tomato bacterial wilt by exploiting these disease-related spectral signatures.

### 4.5 Limitations and future perspectives

Despite the promising performance of the proposed CWT-STNet model in this study, several limitations should be acknowledged. First, although the disease labels were confirmed using the bioluminescent strain *Ralstonia solanacearum* FJ91-LUX, accurate annotation of early infection stages (Levels 1-2) remains inherently challenging because pathogen colonization occurs before visible symptoms develop. Consequently, the temporal boundary between adjacent early disease stages may still involve a certain degree of uncertainty. Second, the CWT-STNet model was developed and evaluated under controlled experimental conditions using a single tomato cultivar, and the available dataset was relatively limited for deep learning applications. Therefore, further validation using larger datasets collected from multiple cultivars, different growth stages, and diverse environmental conditions is necessary to improve the robustness and generalization capability of the model. It would help improve the robustness and generalization capability of the proposed CWT-STNet model to use larger datasets collected from multiple cultivars, different growth stages, and diverse environmental conditions. Moreover, while CWT-STNet demonstrated high performance in detecting the tomato bacterial wilt, its attention mechanism may exhibit response bias when faced with the common problem of overlapping stress phenotypes in the field (such as Fusarium wilt and bacterial wilt-like symptoms caused by drought/salt stress). To enhance the practical application potential of this framework, it is necessary to incorporate the aforementioned biotic and abiotic stresses as negative samples into the training and testing system to quantify the confidence decay of the model under mixed scenarios, and based on this, conduct targeted feature decoupling optimization.

## 5 Conclusions

This study developed a novel CWT-STNet framework that integrates continuous wavelet transform with a Transformer network for early-stage detection of tomato bacterial wilt using hyperspectral data. The results demonstrated that CWT effectively enhances weak spectral features associated with early infection, leading to substantial improvements in detection performance for both conventional models and the proposed framework. Among all models, CWT-STNet achieved superior accuracy and robustness, confirming its effectiveness in capturing global spectral dependencies. Furthermore, SHAP-based interpretability analysis successfully identified the most sensitive wavelengths for early infection stages of bacterial wilt in tomato as 869.0 nm, 1168.1 nm, and 1596.1 nm. These wavelengths are correlated with changes in leaf cell structure, the edge of the water absorption band, and chlorophyll content, respectively, indicating that *Ralstonia solanacearum* infection markedly affects the physiological and structural characteristics of plants in the early stages. Overall, the proposed framework offers a reliable and non-destructive approach for early disease detection, with strong potential to support precision agriculture and intelligent disease management in greenhouse tomato production.

## Data Availability

The original contributions presented in the study are included in the article/supplementary material. Further inquiries can be directed to the corresponding author.
